# Ca^2+^ homeostasis maintained by TMCO1 underlies corpus callosum development via ERK signaling

**DOI:** 10.1038/s41419-022-05131-x

**Published:** 2022-08-04

**Authors:** Ke-Yan Yang, Song Zhao, Haiping Feng, Jiaqi Shen, Yuwei Chen, Si-Tong Wang, Si-Jia Wang, Yu-Xin Zhang, Yun Wang, Caixia Guo, Hongmei Liu, Tie-Shan Tang

**Affiliations:** 1 State Key Laboratory of Membrane Biology, Institute of Zoology, University of Chinese Academy of Sciences, Chinese Academy of Sciences, Beijing, 100101 China; 2 Beijing Institute of Genomics, University of Chinese Academy of Sciences, Chinese Academy of Sciences/China National Center for Bioinformation, Beijing, 100101 China; 3 Institute for Stem cell and Regeneration, Chinese Academy of Sciences, Beijing, 100101 China; 4 Beijing Institute for Stem Cell and Regenerative Medicine, Beijing, 100101 China

**Keywords:** Neuroscience, Developmental biology

## Abstract

Transmembrane of coiled-coil domains 1 (TMCO1) plays an important role in maintaining homeostasis of calcium (Ca^2+^) stores in the endoplasmic reticulum (ER). TMCO1-defect syndrome shares multiple features with human cerebro-facio-thoracic (CFT) dysplasia, including abnormal corpus callosum (CC). Here, we report that TMCO1 is required for the normal development of CC through sustaining Ca^2+^ homeostasis. *Tmco1*^−/−^ mice exhibit severe agenesis of CC with stalled white matter fiber bundles failing to pass across the midline. Mechanistically, the excessive Ca^2+^ signals caused by TMCO1 deficiency result in upregulation of FGFs and over-activation of ERK, leading to an excess of glial cell migration and overpopulated midline glia cells in the indusium griseum which secretes Slit2 to repulse extension of the neural fiber bundles before crossing the midline. Supportingly, using the clinical MEK inhibitors to attenuate the over-activated FGF/ERK signaling can significantly improve the CC formation in *Tmco1*^−/−^ brains. Our findings not only unravel the underlying mechanism of abnormal CC in TMCO1 defect syndrome, but also offer an attractive prevention strategy to relieve the related agenesis of CC in patients.

## Introduction

The corpus callosum (CC) is the largest connective structure in the mammalian brain which comprises over 190 million of axons that transfer information between the hemispheres [1]. It controls integration of sensory, motor information and elaboration of coordinated behavior [2, 3]. A rare disorder, agenesis of the corpus callosum (AgCC), which fails to develop the fibers that connect the hemispheres, occurs in 1:4000 individuals [4]. Abnormalities of the CC have been reported to be associated with a number of neurodevelopmental disorders such as autism spectrum disorder, attention-deficit/hyperactivity disorder, and schizophrenia [5–7].

The formation of CC relies on the coordination of a cascade of critical developmental events. These include the patterning and formation of midline glia structures, the generation and specification of the callosal neurons and their axons, and navigation and growth of these axons across the midline to innervate their final targets in the contralateral hemisphere [8]. Most of the commissural projection neurons cross the midline via CC which are called callosal projection neurons (CPN) [9]. When stimulated by multiple guidance cues including semaphrins, ephrins, fibroblast growth factors, netrins, and slits at the corticoseptal boundary region, growing axons of CPNs extend across the midline and reach their targets in the contralateral hemisphere [8, 10–13]. Defects in CPNs, midline patterning, guidance cues expression and reception result in either partial or complete AgCC [11, 14–16].

Calcium ion (Ca^2+^) is a highly versatile intracellular signal that controls many different cellular functions [17, 18]. Maintaining Ca^2+^ homeostasis in Ca^2+^ stores is pivotal for proper Ca^2+^ signaling and cellular functions [18–30]. Transmembrane and coiled-coil domains 1 (TMCO1) is a highly conserved membrane protein resided in the endoplasmic reticulum (ER). TMCO1 works as a Ca^2+^ load-activated Ca^2+^ (CLAC) channel to prevent ER Ca^2+^ from overfilling, thus maintaining the Ca^2+^ homeostasis in ER [31]. Frameshift mutations in *Tmco1* gene can cause an autosomal-recessive TMCO1-defect syndrome, characterized by distinctive craniofacial skeletal anomalies, mental retardation, ataxia, and many other clinical symptoms [32–34]. The syndrome now belongs to the genetically heterogeneous cerebrofaciothoracic (CFT) dysplasia spectrum [35–38]. Interestingly, AgCC can be found in more than half of the TMCO1 defect patients by MRI studies [32, 36, 37]. However, the mechanism(s) underlying AgCC and the developmental basis of CC abnormalities in TMCO1 defect patients remain unknown.

In this study, we found that severe AgCC occurred in *Tmco1* KO mice, reproducing the main neuropathological feature of TMCO1-defect patients. Upregulation of fibroblast growth factors (FGFs) expression and abnormal translocation of midline glial cells were observed in the midline structures of *Tmco1*^−/−^ brains, resulting in the overpopulated glial cells at indusium griseum (IG) region and halted extension of neural fiber bundles just before crossing the midline. Intriguingly, we found that the abnormal midline glial cell structures were attributed to the increased FGFs and the hyperactive extracellular signal-regulated protein kinase (ERK) pathway caused by supernormal Ca^2+^ signaling. Pharmacological suppression of MEK components via clinical MEK inhibitors (MEKi) were able to rebuild the midline glial cell structures and restore the development of CC in *Tmco1* mutants.

## Results

### TMCO1 deficiency impairs the corpus callosum formation

**Fig. 1:**
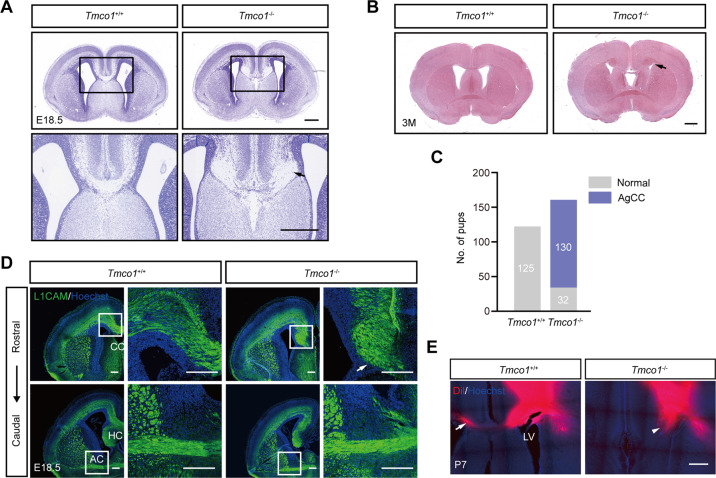
Defects in the formation of the corpus callosum and hippocampal commissure in developing brains of *Tmco1*^−/−^ mice. **A** Nissl staining of coronal sections of *Tmco1*^+/+^ and *Tmco1*^−/−^ brains at E18.5. Lower panels, high magnification of the areas boxed in the upper. Arrow indicates the agenesis of the corpus callosum (AgCC) in *Tmco1*^−/−^ brains. Scale bar, 500 μm. **B** H&E staining of coronal sections of *Tmco1*^+/+^ and *Tmco1*^−/−^ brains at 3 months old. Arrow indicates the abnormal corpus callosum in *Tmco1*^−/−^. Scale bar, 1000 μm. **C** Quantification of the number of pups with the AgCC phenotype in *Tmco1*^+/+^ and *Tmco1*^−/−^. Gray indicates normal CC while purple indicates AgCC. **D** L1CAM immunofluorescence (green) in *Tmco1*^+/+^ and *Tmco1*^−/−^ embryos at E18.5 in rostral and caudal coronal sections showing the developing CC region, hippocampal commissure and anterior commissure. High magnification of the areas boxed are shown on the right. Arrow indicates the AgCC. Scale bar, 200 μm. CC corpus callosum, HC hippocampal commissure, AC anterior commissure. **E** Callosal axons projection pattern monitored by DiI crystals (red), which was injected in the cortex of one hemisphere and allowed to diffuse via callosal axons to the contralateral hemisphere in P7 *Tmco1*^+/+^ or *Tmco1*^−/−^ mice. Scale bar, 500 μm. Arrow, the correctly projected callosal axons to the contralateral hemisphere in *Tmco1*^+/+^; arrowhead, Probst bundles formed in *Tmco1*^−/−^. LV lateral ventricle. See also Fig. S1.

Corpus callosum is the largest commissural tract connecting the two cerebral hemispheres [39]. We found that *Tmco1* deletion in mice resulted in severe AgCC in perinatal period (Fig. 1A), which can sustain to adult stage (Fig. 1B). Consistent with the patient clinical feature of TMCO1-defect syndrome, about eighty percent (130/162) of the *Tmco1*^−/−^ mice exhibited complete AgCC (Fig. 1C). While the cell adhesion molecule L1 (L1CAM)-positive callosal axons successfully crossed the midline in the wild-type (WT) brains at E18.5, their extension in the *Tmco1*^−/−^ brains halted/accumulated on both sides and formed the Probst bundles (PB) (Fig. 1D, arrow). In addition, we observed that these embryos also lacked the hippocampal commissure (HC) with the anterior commissure (AC) unaffected (Fig. 1D). To further investigate callosal axons defects in the *Tmco1*^−/−^ mice, we performed axonal tracing experiment by labeling DiI crystals which were injected into the cortex of one hemisphere of P7 mice. In contrast to the WT, whose callosal axons had successfully crossed the midline and extended to the contralateral side (Fig. 1E, arrow), DiI-labeled axons in the *Tmco1*^−/−^ could not be detected in the contralateral hemisphere (Fig. 1E, arrowhead), representing a complete AgCC in *Tmco1*^−/−^ brains.

To understand how TMCO1 deficiency impairs the CC formation, we determined the expression pattern and dynamic changes of TMCO1 in the telencephalon from E13.5 to P28. In situ hybridization (ISH) for *Tmco1* revealed that *Tmco1* was lowly expressed in the dorsal pallium at E13.5, with increased expression throughout the ventricular zone /subventricular zone (VZ/SVZ) region at E15.5 (Fig. S1A). Interestingly, when the CC begins to develop between E15.5 and E17.5, the primary regions of *Tmco1* expression shifted from the VZ/SVZ to the neocortex and midline structures such as indusium griseum (IG) and glia wedge (GW) (Fig. S1A), corresponding to the CC position. By contrast, no specific signal was observed with the control probe (Fig. S1B). Quantitative RT-PCR and western-blotting of embryonic brains also revealed that the transcript and protein abundance of TMCO1 exhibited dynamic changes during E13.5–P28, with a maximum level around E15.5–E17.5 (Fig. S1C–S1E), a stage in coincidence with the CC formation. Therefore, the temporal and spatial expression profiles of *Tmco1* further support a key role of TMCO1 in CC formation.

### Deletion of *Tmco1* deranges the development of midline glial structures

The axons of CPNs connect the two hemispheres via the corpus callosum. It is known that mouse CPNs reside in neocortical layers II/III (~80%), V (~20%), and VI. To further explore the underlying mechanisms of AgCC in *Tmco1*^−/−^ mice, we performed immunostaining using the layer-specific markers, with SATB2 for CPNs in all layers of cortex, CTIP2 for subcerebral projection neurons of layer V and TBR1 for layer VI, respectively [9, 13, 40]. We found that TMCO1 deficiency did not alter the expression and distribution of all markers, indicating that CPNs were specified correctly (Fig. S2A and S2B). Neurite outgrowth is crucial for neural and corpus callosum development [41, 42]. In the preparatory phase of the corpus callosum formation, there was no significant difference in the midline structure between *Tmco1*^+/+^ and *Tmco1*^−/−^ at E14.5 (Fig. S2C), and the histological results showed that the axons from cingulate gyrus and cortex could extend to reach the midline at E15.5 (Fig. S2C, arrowheads), suggesting that the CPN axon development and extension were not significantly affected by *Tmco1* depletion. The connection between the two hemispheres––the corpus callosum, could not be formed in *Tmco1*^−/−^ mice at E16.5 (Fig. S2C, arrow), with the PB-like structure in *Tmco1*^−/−^ brains (Fig. S2C, arrow) reflecting an accumulation of stalled neural fibers before crossing the midline. These data suggest that agenesis of CC in *Tmco1*^−/−^ mice may not be due to the defect in the axon growth of CPNs. To further test whether TMCO1 deficiency affects the neurite outgrowth, we performed in vitro analyses in the primary cultures of cortical neurons. The neurites were labelled by immunofluorescence staining of TuJ1 at DIV3 and DIV7. No significant differences were observed in the length of the longest neurites at DIV3 and DIV7 (Fig. S2D and S2E), and in the neurite complexity at DIV7 (Fig. S2F) between cortical neurons of *Tmco1*^+/+^ and *Tmco1*^−/−^. We then speculated that non-CPN autonomous factors might be involved in this AgCC phenotype.

**Fig. 2:**
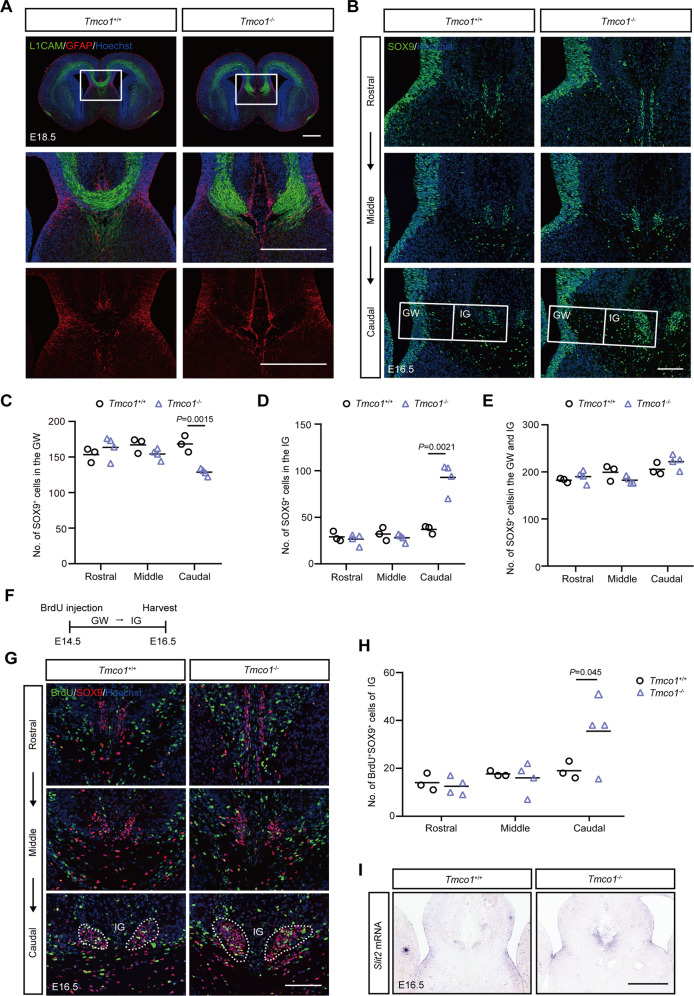
TMCO1 deficiency increases the migration of glial cells from the GW to the IG. **A** Upper: Immunofluorescence for the axonal marker LICAM (green) and the glial marker GFAP (red) in coronal sections of *Tmco1*^+/+^ and *Tmco1*^−/−^ embryonic brains at E18.5. High magnification of the midline area (white box) is shown in lower panels. Scale bar, 500 μm. **B** SOX9 immunofluorescence (green) at the telencephalic midline in *Tmco1*^+/+^ and *Tmco1*^−/−^ embryonic brains in rostral, middle and caudal coronal sections through the developing CC region. Scale bar, 100 μm. GW glia wedge, IG indusium griseum. **C**–**E** The counts of SOX9-positive cells in the GW (**C**), IG (**D**) and GW + IG (**E**). *Tmco1*^+/+^, *n* = 3; *Tmco1*^−/−^, *n* = 4. Two-tailed unpaired Student’s *t* test. **F** The timeline of the experiment for SOX9 immunostaining after BrdU injection. **G** BrdU (green) and SOX9 (red) co-immunofluorescence at the telencephalic midline of *Tmco1*^+/+^ and *Tmco1*^−/−^ embryonic brains at E16.5 after a single BrdU administration at E14.5. Scale bar, 100 μm. IG indusium griseum. (**H**) Quantification of the number of BrdU^+^SOX9^+^ cells at the IG in *Tmco1*^+/+^ and *Tmco1*^−/−^ midline. *Tmco1*^+/+^, *n* = 3; *Tmco1*^−/−^, *n* = 4. Two-tailed unpaired Student’s *t* test. (**I**) In situ hybridization for the expression of *Slit2* in coronal sections from *Tmco1*^+/+^ and *Tmco1*^−/−^ E16.5 embryos. Scale bar, 500 μm. See also Figs. S2 and S3.

Multiple glial structures, such as the glia wedge (GW), midline zipper glia (MZG) and indusium griseum (IG), are required for the formation of CC, which can be marked by GFAP immunohistochemistry as early as E13 (GW) and E17 (MZG and IG) [11, 43]. The GFAP-positive cells in the IG and MZG were notably disorganized in *Tmco1*^−/−^ mice (Fig. 2A). Since the disorganization of the midline glial cells could be a secondary effect of callosal axon misprojection, we analyzed the glial cells phenotypes during CC formation by SOX9, a nuclear marker to quantify these cells in IG and GW along rostral to caudal axis [44, 45]. In *Tmco1*^−/−^ telencephalon at E16.5, there were very few SOX9-positive cells in the IG region rostrally, while strikingly increased number of SOX9-positive cells could be detected in caudal sections which was about 2-fold higher than that in the WT embryos (Fig. 2B, D). Notably, the SOX9-positive area in the GW of *Tmco1*^−/−^ was getting thinner when it became enlarged in the IG at E16.5 (Fig. 2B, C). Nevertheless, the total number of SOX9-positive cells in GW and IG complex along the rostro-caudal axis displayed no significant difference relative to WT (Fig. 2B, E), indicating that TMCO1 deficiency does not disturb the size of midline progenitor pool. Since the glial cells of IG are derived from radial glial progenitor detaching their apical foot from the GW and translocating their nuclei toward IG [46], this prompted us to determine whether there was an excessive GW to IG translocation of glia cells in the *Tmco1*^−/−^ telencephalon. Given that most cells of IG were born around E14.5 [43], we labeled dividing glial cells in GW and IG trip at E14.5 by a single BrdU injection (Fig. 2F) and tracked their fate and location in the midline glia structures of both WT and *Tmco1* mutants. By E16.5, in the *Tmco1* mutants, significantly more BrdU and SOX9 double-labeled glial cells were seen in the IG of caudal sections (Fig. 2G, H), confirming an excess of glia cell translocation from GW to IG in *Tmco1*^−/−^ brains. Together, our data showed that, relative to the WT counterparts, abnormally increased numbers of glial cells in the IG region accompanied by a concomitant depletion of the GW glia pool in *Tmco1*^−/−^ embryos were stemmed from the excessive glial cell migration from GW to IG.

It is known that Slit2, secreted by the glia cells of GW and IG, is an indispensable chemorepulsive ligand to steer axon guidance in the midline [47], whose imbalanced gradient can lead to AgCC [48, 49]. Our ISH assay showed that, due to the overpopulated glia cells in the IG region, the *Slit2* expression area was significantly expanded and formed a compact focus in the IG of E16.5 *Tmco1*^−/−^ brains (Fig. 2I), causing the imbalanced gradient of Slit2 signal between GW and IG. We further examined the involvement of other major developmental ligand/receptor systems which had been previously reported to be associated with corpus callosum formation, such as netrin, semaphorin, and ephrin families [50–52]. No change in the expression patterns for these axon guidance factors and receptors was found in *Tmco1*^*−/−*^ mouse brains (Fig. S3).

Combined, these data demonstrate an excess of glial cell population migrating from GW to IG in *Tmco1* mutants, which can further derange the normal midline glial structure and gradient balance of Slit2 signal, thereby disrupting the navigation of callosal axons.

### Ca^2+^-dependent upregulation of FGF8/17 and ERK signaling in the midline of *Tmco1*^−/−^ brain

**Fig. 3:**
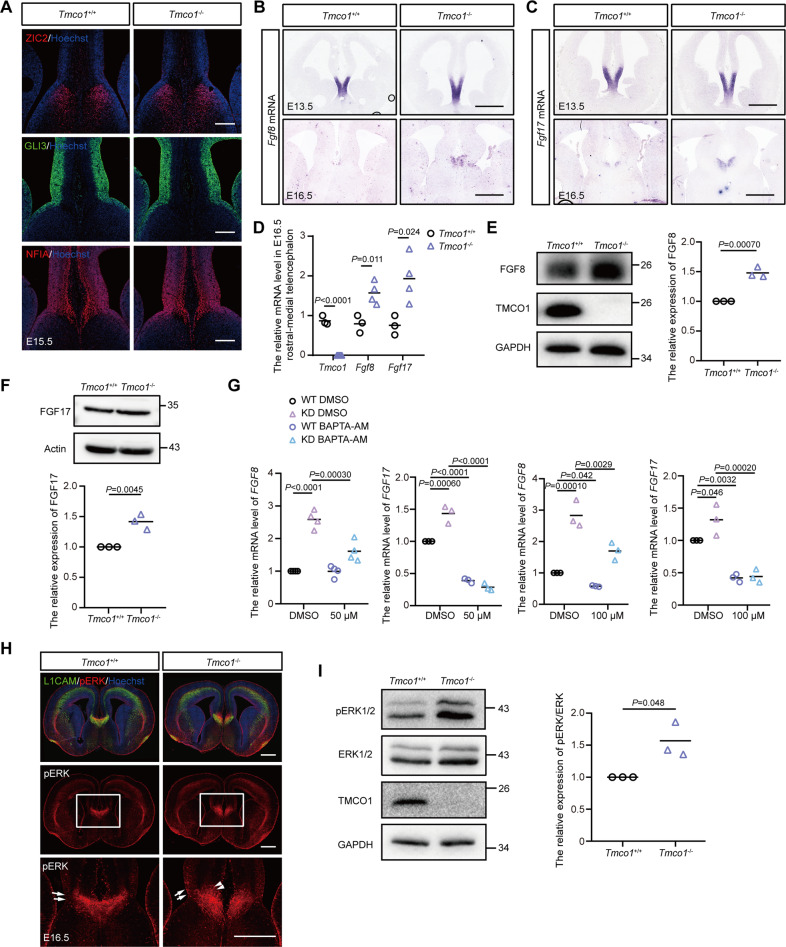
TMCO1 depletion upregulates FGF8/17 and over-activates ERK signaling in the *Tmco1* mutants. **A** Immunofluorescence of the factors required for the formation of commissural plate and corticoseptal boundary (ZIC2, GLI3, NFIA) in E15.5 brains of *Tmco1*^+/+^ and *Tmco1*^−/−^. Scale bar, 200 μm. **B**, **C** In situ hybridization analyses of *Fgf8* (**B**) and *Fgf17* (**C**) in E13.5 and E16.5 brains of *Tmco1*^+/+^ and *Tmco1*^−/−^. Scale bar, 500 μm. **D** qRT-PCR analysis of *Tmco1*, *Fgf8* and *Fgf17* expression in the E16.5 rostral-medial telencephalons of *Tmco1*^+/+^ and *Tmco1*^−/−^. *Tmco1*^+/+^, *n* = 3; *Tmco1*^−/−^, *n* = 4. Two-tailed unpaired Student’s *t* test. **E** Western-blotting analysis of FGF8 extracted from E16.5 rostral-medial brains of *Tmco1*^+/+^ and *Tmco1*^−/−^. GAPDH is used as a loading control. Right panel, Relative quantification of western blotting analysis of FGF8 protein levels in the rostral-medial brain of *Tmco1*^+/+^ and *Tmco1*^−/−^. Data is represented as the mean protein intensity normalized to GAPDH. *Tmco1*^+/+^, *n* = 3; *Tmco1*^−/−^, *n* = 3. Two-tailed unpaired Student’s *t* test. **F** Western-blotting analysis of FGF17 extracted from E16.5 rostral-medial brains of *Tmco1*^+/+^ and *Tmco1*^−/−^. Actin is used as a loading control. Lower panel, Relative quantification of western blotting analysis of FGF17 protein levels in the rostral-medial brain of *Tmco1*^+/+^ and *Tmco1*^−/−^. Data is represented as the mean protein intensity normalized to Actin. *Tmco1*^+/+^, *n* = 3; *Tmco1*^−/−^, *n* = 3. Two-tailed unpaired Student’s *t* test. **G** The expression of *Fgf8* and *Fgf17* were up-regulated in TMCO1 deficient cells in a Ca^2+^-dependent manner. qRT-PCR analysis of *Fgf8* and *Fgf17* expression in the wild-type (WT) or *Tmco1*-KD HeLa cells treated with/without 50 or 100 μM BAPTA-AM. One-way ANOVA with Tukey’s correction for multiple comparisons. (**H**) pERK1/2 immunohistochemistry on E16.5 coronal sections from *Tmco1*^+/+^ and *Tmco1*^−/−^ brains. Arrows, pERK1/2-positive cells in GW. Arrowheads, pERK1/2-positive cells in IG. High magnification of the areas boxed are shown in lower panels. Scale bar, 500 μm. **I** Western-blotting analysis of proteins extracted from E16.5 whole telencephalons of *Tmco1*^+/+^ and *Tmco1*^−/−^. Right panel, relative quantification of western blotting analysis of pERK/ERK in the telencephalons of *Tmco1*^+/+^ and *Tmco1*^−/−^. *Tmco1*^+/+^, *n* = 3; *Tmco1*^−/−^, *n* = 3. Two-tailed unpaired Student’s *t* test.

After determining that midline glial structure defects were responsible for the phenotype of AgCC in *Tmco1* mutants, we next examined the early pattern in the corticoseptal boundary where midline structures formed later [53]. The expression patterns of ZIC2 and GLI3, NFIA (the factors required for the formation of commissural plate and corticoseptal boundary) were unaffected in *Tmco1* mutants at E15.5 (Fig. 3A). FGF signaling is known to be essential for the formation of commissural plate [54], with the FGF8 and FGF17 regulating the translocation of glial cells from GW to IG [15, 46, 55]. We found that the transcripts of *Fgf8* and *Fgf17* were confined to the commissural plate, but significantly expanded further dorsally in the E13.5 *Tmco1* mutants (Fig. 3B, C). After CC formation at E16.5, the expressions of *Fgf8*/*17* were still much higher in the IG of *Tmco1*^−/−^ brains (Fig. 3B, C). qRT-PCR analysis and western-blotting using rostral-medial brain tissues at E16.5 showed that the expression of *Fgf8* and *Fgf17* increased in *Tmco1* mutants (Fig. 3D–F). Upregulation of *Fgf8*/*17* expression was also detected in *Tmco1*-knockdown (KD) HeLa cells, which could be significantly blocked by a well-known Ca^2+^ chelator BAPTA-AM (Fig. 3G), indicating a Ca^2+^-dependent upregulation of *Fgf8*/*17* expression at commissural plate.

ERK signaling in the developing brain is driven by FGF signaling [56]. Therefore, we next examined if the ERK signaling pathway is over-activated during CC formation in *Tmco1*^−/−^ telencephalon. In *Tmco1*^−/−^ embryonic brains, there was a global increase in the level of pERK1/2 (Fig. 3H). Immunofluorescence staining showed that pERK1/2-positive signal could be detected in some GW (Fig. 3H, arrow) and IG (Fig. 3H, arrowhead) cells in both WT and mutants. Interestingly, there were more pERK1/2 cells with higher level of pERK1/2 at the *Tmco1*^−/−^ brain midline structure than WT counterparts, especially in IG region (Fig. 3H). Increased phosphorylation of ERK1/2 in the E16.5 *Tmco1*^−/−^ telencephalon was further confirmed by western-blotting (Fig. 3I). Therefore, the ERK signaling is apparently over-activated especially in the midline glial structures in *Tmco1*^−/−^ brains.

**Fig. 4:**
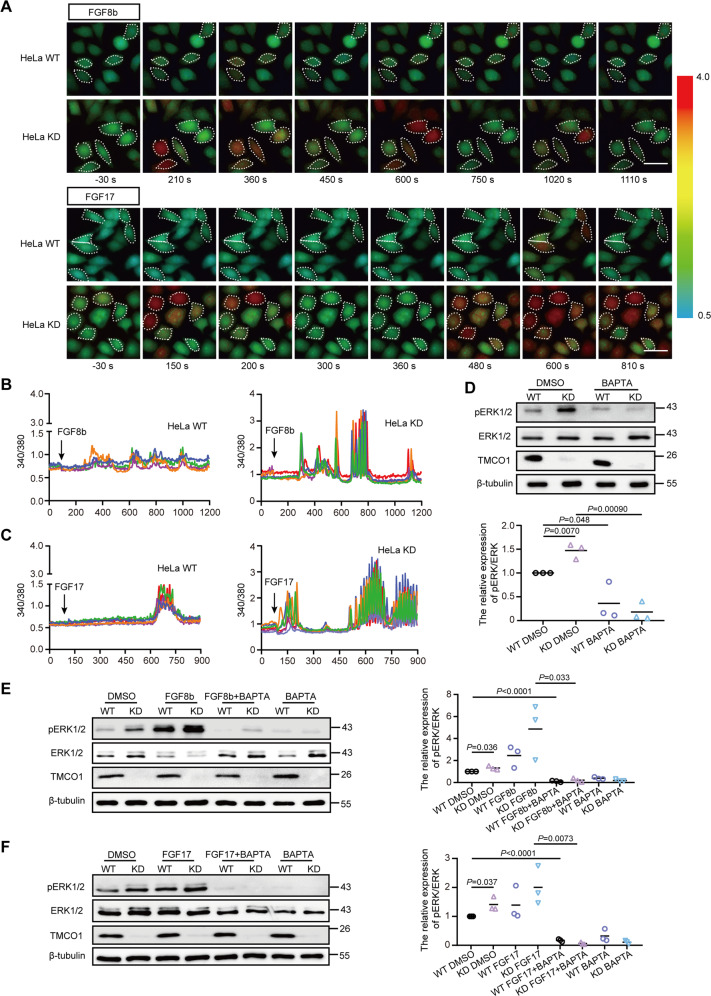
TMCO1 deficiency causes overactive ERK signaling upon FGFs stimulation, which related to supernormal Ca^2+^ signaling in vitro. **A** Fura-2 ratio (340/380) was used to indicate the cytosolic Ca^2+^ concentration. Images show Fura-2 340/380 ratios in wild-type (WT) and *Tmco1*-knockdown (KD) HeLa cells. The pseudo-color calibration scale for 340/380 ratio is shown on the right. Ratios are recorded for 50 ng/ml FGF8b stimulation (upper panel) or FGF17 stimulation (lower panel) in WT and *Tmco1* KD HeLa cells. Upper panel, 340/380 ratio images are shown for cells 30 s before, and 210 s, 360 s, 450 s, 600 s, 750 s, 1020 s and 1110 s after stimulation of 50 ng/ml FGF8b. Lower panel are shown for cells 30 s before, and 150 s, 200 s, 300 s, 360 s, 480 s, 600 s and 810 s after stimulation of 50 ng/ml FGF17. Scale bar, 20 μm. **B**, **C** FGF8b (**B**) and FGF17 (**C**) -evoked Ca^2+^ transients in WT and *Tmco1*-KD HeLa cells in HBSS medium. Compared to WT cells, *Tmco1*-KD cells exhibit supernormal Ca^2+^ signaling with more Ca^2+^ spikes and higher Ca^2+^ spike amplitudes. **D** Western-blotting analysis of proteins extracted from WT or *Tmco1*-KD HeLa cells treated with/without 50 μM BAPTA-AM. β-tubulin is used as a loading control. The relative expression of pERK/ERK is shown in lower panel. *n* = 3. One-way ANOVA with Tukey’s correction for multiple comparisons. **E**, **F** Western-blotting analysis of proteins extracted from WT or *Tmco1*-KD HeLa cells treated with FGF8b (**E**) or FGF17 (**F**) together with/without 50 μM BAPTA-AM. β-tubulin is used as a loading control. The relative expression of pERK/ERK is shown in right panel. *n* = 3. One-way ANOVA with Tukey’s correction for multiple comparisons.

FGFs are known to bind tyrosine kinase receptors to mediate cytoplasmic Ca^2+^ waves by releasing Ca^2+^ from intracellular stores and Ca^2+^ influx at the plasma [57, 58]. As an ER Ca^2+^ channel, TMCO1 defect causes overfilling of ER Ca^2+^ store and supernormal Ca^2+^ signaling [31]. We next examined whether over-activation of the ERK signaling is related to the abnormal Ca^2+^ signaling caused by *Tmco1* deletion. The exogenous stimulation of FGFs were applied to WT and *Tmco1*-KD HeLa cells. Intriguingly, in response to the same concentration of FGF8b or FGF17, while WT cells had one or several Ca^2+^ spikes, KD cells exhibited a supernormal Ca^2+^ signaling (with more Ca^2+^ spikes and higher Ca^2+^ spike amplitudes) (Fig. 4A–C). To evaluate the contribution of Ca^2+^ in ERK signaling activation, cells were treated with BAPTA-AM to chelate the intracellular Ca^2+^ after the addition of FGFs. Our results showed that BAPTA-AM not only abolished the increased basal level of pERK1/2 in KD cells (Fig. 4D), but also blocked FGF8b and FGF17-induced elevation of pERK1/2 in WT and KD cells (Fig. 4E, F), indicating a Ca^2+^-dependent over-activation of ERK signaling in *Tmco1* mutants.

These data demonstrate that intracellular Ca^2+^ signal is essential for the transcription of FGFs and activation of ERK signaling, and that supernormal Ca^2+^ signals lead to upregulation of FGF8/17 and over-activation of ERK in TMCO1 defect cells. Since ERK signaling can regulate the translocation of glial cells from the GW to the IG, over-activation of FGF/ERK pathway is expected to stimulate the excessive migration of glia to the midline IG region in *Tmco1*^−/−^ brains.

### MEK inhibitors relieve the AgCC defect in *Tmco1* mutants

Our above results support that supernormal Ca^2+^ signals in *Tmco1* mutants can cause over-activation of ERK signaling pathway, which in turn lead to excessive migration and overpopulation of glial cells in the IG, causing an imbalanced gradient of Slit2 between GW and IG, thus disturbing the callosal projection axons crossing the midline. We speculated that suppressing the FGF/ERK pathway transiently during CC formation might be able to inhibit the excessive translocation of glia cells to midline IG and ameliorate the AgCC phenotypes.

**Fig. 5:**
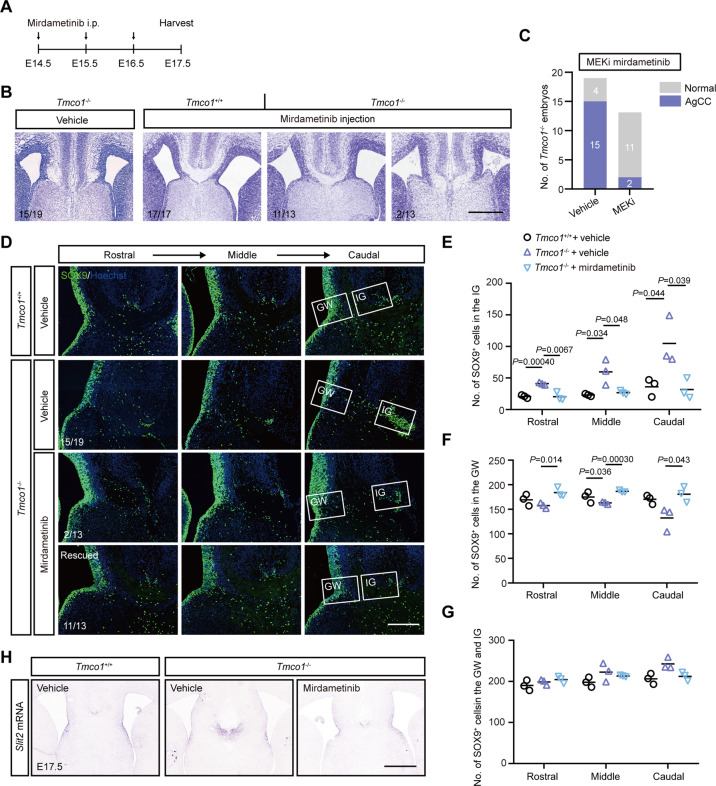
The phenotype of AgCC can be efficiently relieved by MEK inhibitor - mirdametinib. **A** The timeline of mirdametinib injection, which was administrated to pregnant females daily between E14.5 and E16.5 spanning the period of CC formation and harvested at E17.5. **B** Nissl staining of the coronal sections in the *Tmco1*^+/+^ and *Tmco1*^−/−^ treated with/without mirdametinib. Numbers at the bottom left indicate the proportions of embryonic brains with phenotype shown in that panel. Scale bar, 500 μm. **C** Quantification of the number of *Tmco1*^−/−^ embryos showing normal or AgCC phenotypes with/without mirdametinib treatment (i.p., 3.0 mg/kg). Vehicle-injected pregnant females, *n* = 12; mirdametinib-injected pregnant females, *n* = 10. **D** SOX9 immunofluorescence (green) at the telencephalic midline in *Tmco1*^+/+^ and *Tmco1*^−/−^ E17.5 embryos treated with/without mirdametinib in rostral, middle and caudal coronal sections through the developing CC region. Numbers at the bottom left indicate the proportions of embryos with phenotype shown in that panel. Scale bar, 200 μm. **E**–**G** The counts of SOX9-positive cells in the GW (**E**), IG (**F**) and GW + IG (**G**). The vehicle group: *Tmco1*^+/+^, *n* = 3; *Tmco1*^−/−^, *n* = 3; the mirdametinib-treated group: *Tmco1*^−/−^, *n* = 3 (2/3 rescued and 1/3 not fully recovered). One-way ANOVA with Tukey’s correction for multiple comparisons. **H** In situ hybridization for the expression of *Slit2* in the IG of coronal sections from E17.5 *Tmco1*^+/+^ and *Tmco1*^−/−^ embryos treated with/without mirdametinib. Scale bar, 500 μm. See also Fig. S4.

Mirdametinib (PD0325901) is a potent inhibitor of MEK/ERK pathway [45, 59] and has been proved to be effective in treating adult neurofibromatosis type 1 patients [60]. To test our hypothesis, we treated pregnant females daily through intraperitoneal injection with mirdametinib between E14.5 and E16.5 spanning the developmental period for CC formation, and harvested the embryos at E17.5 (Fig. 5A). Our results showed that, while most *Tmco1*^−/−^ brains displayed a complete AgCC phenotype (15/19 embryos, vehicle injection), majority of *Tmco1*^−/−^ embryos (11/13 embryos) collected from the mirdametinib-injected pregnant females exhibited almost normal U-shape CC (Figs. 5B, C, and S4A). Next, we determined if the overpopulated glial cells at the midline IG in *Tmco1*^−/−^ brains could be rescued by mirdametinib. Overpopulated glia cells in *Tmco1*^−/−^ IG were seen in vehicle control group at E17.5 (Fig. 5E), same as presented at E16.5 (Fig. 2B, D). Interestingly, after mirdametinib treatment, the number of SOX9-positive cells was significantly decreased in the IG (Fig. 5D, E), but increased in the GW (Figs. 5D, F), leading to an overall similar count of SOX9-positive cells in GW and IG compartments in *Tmco1*^−/−^ brains as those in WT brains (Fig. 5E–G), showing that mirdametinib treatment can successfully rebuild the midline glial structure. Moreover, mirdametinib treatments not only inhibited the excessive migration of glial cells from GW to IG, but also robustly restored the expression pattern of *Slit2* in the midline structure of *Tmco1*^−/−^ brains (Fig. 5H).

We also used another MEKi - selumetinib (AZD6244) [59, 61] to further validate our observations. Selumetinib was administrated intragastrically from E13.5 to E16.5 (Fig. S4B). We noticed that, while 77% (10/13) of *Tmco1*^−/−^ brains showed complete AgCC, 75% (9/12) of the selumetinib-treated *Tmco1*^−/−^ brains showed normal U-shape CC (Fig. S4C and S4D), demonstrating that AgCC in *Tmco1*^−/−^ brain could also be rescued by selumetinib. *Tmco1* mutants that did not develop complete AgCC displayed a thinner CC than WT (Fig. S4E). Notably, MEK inhibitors not only relieved the AgCC phenotype, but also restored the thickness of CC in those *Tmco1*^−/−^ brains (Fig. S4E).

Collectively, these results show that clinical MEK inhibitors are able to efficiently relieve AgCC phenotype in *Tmco1*^−/−^ brains.

## Discussion

**Fig. 6:**
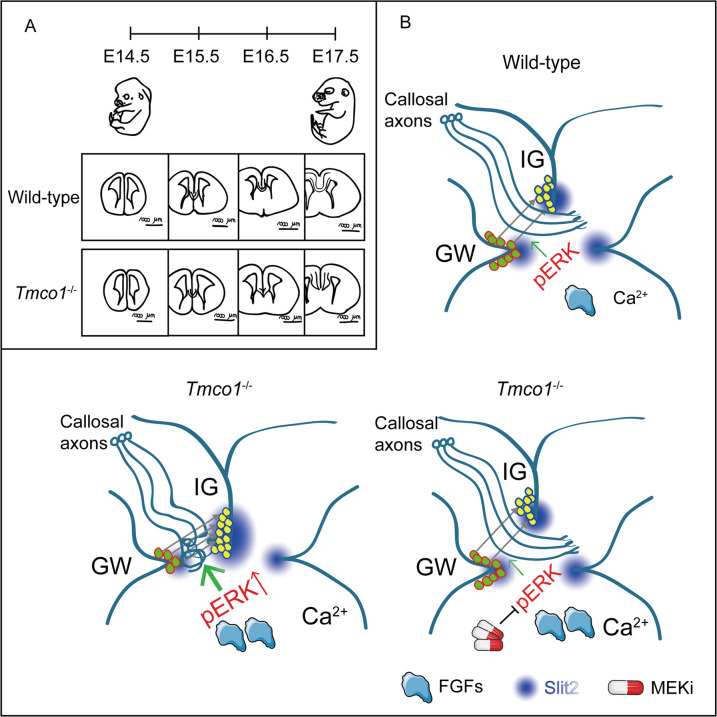
Model for TMCO1 deficiency-induced agenesis of corpus callosum (AgCC) and MEKi-restored corpus callosum (CC) formation. **A** The *Tmco1*^−/−^ brains exhibit severe CC defect during E14.5-E17.5. **B** Compared to wild-type embryonic brains with normal CC extension (**B**, upper right), the abnormal Ca^2+^ homeostasis induced by TMCO1 deficiency leads to supernormal Ca^2+^ signaling, over-activation of the FGF/ERK signaling at the midline, excessive migration of glial cells from GW to IG, and overpopulation of glial cells in the IG (**B**, lower left). Then the robust Slit2 signals secreted by IG glial cells repulse the callosal axon navigation before crossing the midline, leading to a halted extension of callosal axons and severe AgCC (**B**, lower left). Clinical MEK inhibitors efficiently reduce the excessive migration of glial cells from GW to IG, rebuild the Slit2 gradient balance between GW to IG, and restore the normal CC extension across the midline in *Tmco1*^−/−^ brains (lower right). These findings highlight a novel role of Ca^2+^ homeostasis maintained by TMCO1 in orchestrating the midline glial structure and CC formation during embryonic neurodevelopment, and provide a promising prevention strategy to relieve the related AgCC in patients.

More than half of the TMCO1 defect patients have complete or partial AgCC. However, the underlying mechanism(s) and the developmental basis of CC abnormalities remain unknown. Here, we show that deletion of *Tmco1* gene (*Tmco1*^−/−^) in mouse causes severe developmental defects of the CC during E14.5-E17.5 (Fig. 6A). Compared to WT embryonic brains (Fig. 6B, upper right), excessive glial cell migration from GW to IG and overpopulated glia in IG were observed in *Tmco1*^−/−^ brains, causing the disorganization of midline glial structure and imbalanced Slit2 gradient between GW and IG (Fig. 6B, lower left). We further demonstrate that it is the dysregulation of Ca^2+^ homeostasis induced by TMCO1 deficiency over-activates the FGF/ERK signaling pathway at the midline (increased expression of FGF8/17 and hyperactive ERK signaling), which promotes an excess of glial cell migration from GW to IG and overpopulation of glial cells in the IG (Fig. 6B, lower left). Then the robust Slit2 signals secreted by IG glial cells repulse the callosal axon navigation before crossing the midline, leading to a halted extension of callosal axons and severe AgCC (Fig. 6B, lower left). Clinical MEK inhibitors efficiently reduce the excessive migration of glial cell from GW to IG, rebuild the Slit2 gradient balance between GW to IG, and restore the normal CC formation in *Tmco1*^−/−^ brains (Fig. 6B, lower right). These findings highlight a novel role of Ca^2+^ homeostasis maintained by TMCO1 in orchestrating the midline glial structure and CC formation during embryonic neurodevelopment, and provide a promising prevention strategy to relieve the related AgCC in patients.

FGF signaling was reported to regulate the translocation of glia cells from the GW to the IG [15, 45, 46]. *Fgf8* and *Fgf17* are expressed at the anterior patterning center of telencephalon during early embryogenesis and later at the midline within the presumptive commissural plate [62, 63]. Abolishing the FGF receptor function prevents glial cells from reaching the pial surface and forming the IG structure. Conversely, implanting FGF8 or FGF17-soaked beads into the cerebral cortex induces excessive translocation of radial glia cells to the side of FGF-soaked beads [15, 46, 55]. In *Tmco1*^−/−^ brains, overpopulated glial cells were observed in the midline IG structure. Our ISH (Fig. 3B, C), qRT-PCR (Fig. 3D) and western-blotting (Fig. 3E, F) results showed that, although no distribution differences of *Fgf8* and *Fgf17* mRNA expression was observed between WT and *Tmco1*^−/−^ brains, there were significant increases of *Fgf8* and *Fgf17* at both mRNA and protein level in *Tmco1*^*−/−*^ brains, indicating that the enhanced expression of FGFs could be a causing factor for overpopulated glial cells in IG region of *Tmco1*^−/−^ brains. Interestingly, the enhanced expression of FGFs was found to be Ca^2+^-dependent, as Ca^2+^ chelation by BAPTA-AM significantly down-regulated the transcription of *FGF8* and *FGF17* (Fig. 3G).

FGF signaling has been implicated in neurite outgrowth and retinal axon guidance [64, 65]. Knockout of *Fgfr1* in the telencephalon led to not only the midline patterning defect but also the commissural axon defect [55]. Mice homozygous for the recombined *Fgfr1* null allele (*Fgfr1*^f/f;hGFAPCre^) lacked the CC and HC, but the conditional knockout of *Fgfr1* in neurons did not produce any detectable phenotype with the normal formation of midline glial structures and CC [46], which suggested the midline structures and axons defect could be two relatively independent processes. In our study, *Tmco1* deletion caused the elevated expression of both *Fgf8* and *Fgf17*, which led to the overpopulation of midline glia in IG and agenesis of CC. *Tmco1* ablation did not cause significant changes in the specification, lamination and neurite outgrowth of cortical neurons (Fig. S2A-S2F), suggesting that agenesis of CC in *Tmco1*^*−/−*^ brains may not be attributed to the defect in axon growth of CPNs.

Overpopulated glial cells in IG may also involve hyperactive ERK signaling in *Tmco1*^−/−^ radial glia cells. The ERK cascade is one of the major intracellular signaling pathway downstream of FGFs. FGFs bind tyrosine kinase receptors to trigger cytoplasmic Ca^2+^ transients which may be important for activation of ERK signaling. Since *Tmco1* deletion caused overload of intracellular stores and supernormal Ca^2+^ signaling (Fig. 4A–C), it is possible that the supernormal Ca^2+^ signaling may result in hyperactive ERK signaling. Indeed, significantly more pERK1/2-positive glia cells with higher level of pERK1/2 were found in the midline structure of *Tmco1*^−/−^ brains (Fig. 3H, I), confirming the hyperactive ERK signaling cascade in these glial cells, which could promote excessive migration of glial cells from GW to IG (Fig. 2B–H) and imbalanced Slit2 signals between GW and IG areas (Fig. 2I). Notably, hyperactive ERK signaling can be abolished by intracellular Ca^2+^ chelation (Fig. 4D–F), demonstrating a Ca^2+^-dependent over-activation of ERK signaling cascade in *Tmco1*^−/−^ brains. Therefore, these data support that ER Ca^2+^ homeostasis and normal Ca^2+^ signaling maintained by TMCO1 are essential to confer proper FGF/ERK signaling, normal midline glial structure, balanced Slit2 signals between GW and IG, and correct callosal axons navigation.

FGF/ERK signaling axis dramatically affects neurodevelopment and function, such as development of the VZ and the corpus callosum [66]. Our results demonstrate that, due to dysregulation of Ca^2+^ homeostasis and supernormal Ca^2+^ signaling, FGF/ERK signaling cascade is over-activated in *Tmco1*^−/−^ glial cells, causing the disorganization of the midline glial structure (overpopulation of glial cells in the IG). It is known that normal midline glia distribution pattern is important for guiding callosal axons and defining navigation route. Multiple gene mutations causing AgCC, such as *RapGEF1*, *Bmp7*, *Vasp*, *Rfx3* and *Gli3* [67–72], are associated with the abnormal midline glia pattern. For the first time, our study established a causative link among dysregulation of Ca^2+^ homeostasis, disorganization of midline glial structure orchestrating, and defect of callosal axon navigation.

Mirdametinib and selumetinib, two selective MEK non-ATP-competitive inhibitors [59], have been proved to be effective to treat the Neurofibromatosis type 1 patients [60, 73]. Selumetinib was approved by Food and Drug Administration (FDA) in May 2020 [60]. Our data from in vivo experiments showed that, AgCC in *Tmco1*^−/−^ brains could be remarkably rescued by transient treatment with mirdametinib and selumetinib, thus providing a prevention/therapeutic strategy to relieve AgCC for TMCO1 defect syndrome. Given that dysregulation of FGF/ERK signaling pathway is associated with multiple human disorders with neurodevelopment defects, such as Kallmann syndrome and RASopathies [74–77], pharmacologically adjusting the activity of FGF/ERK signaling during embryonic neurodevelopment stage may also have prevention/therapeutic value to the affected individuals.

In summary, we discover that Ca^2+^ homeostasis maintained by TMCO1 is a novel regulator for corpus callosum formation. TMCO1 is essential to confer proper Ca^2+^ signaling, proper FGF/ERK signal, normal midline glial structure and correct callosal axon navigation. We also identify a potential prevention/therapeutic strategy for AgCC in TMCO1 defect patients.

## Materials and methods

### Mice

The *Tmco1*^+/−^ mouse strain (C57BL/6 J) was generated in Shanghai Model Organisms Center, Inc. (Shanghai, China), as described previously [78]. Embryos analyzed in this study were of either sex. The morning of the day of the appearance of the vaginal plug was defined as embryonic day (E) 0.5. The day of birth was designated postnatal day (P) 0. All animal experiments were reviewed and approved by the Institute of Zoology Institutional Animal Care and Use Committee and were conducted according to the committee’s guidelines. Mice were raised in a temperature-controlled environment with a 12-h light–dark cycle.

### Immunohistochemistry

Embryonic brains were fixed in 4% (wt/vol) paraformaldehyde (PFA) in phosphate-buffered saline (PBS) at 4 °C overnight, cryoprotected in 30% sucrose and embedded in OCT frozen medium for frozen sectioning. A total of 10 μm coronal sections were processed for immunohistochemistry. Sections were washed in PBS and then blocked for 1 h at room temperature. After blocking, sections were incubated with primary antibodies at 4 °C overnight. Sections were stained with secondary species-specific antibodies conjugated to Alexa-488, Alexa-561, or Alexa-647 (Molecular Probes, Eugene, OR, USA) for 2 h at room temperature, and counterstained with nuclear dye Hoechst 33342. Images from the immunostained brain sections were acquired through Andor Dragonfly 505 and Leica STELLARIS 5. Images were further processed using ImageJ and Photoshop CS6. Please see the Key resources table for the details of all the materials used in this study.

### Axonal tracing

After fixation of the brains in 4% PFA, single crystals of 1,1′-dioctadecyl-3,3,3′,3′-tetramethylindocarbocyanine perchlorate (DiI, Invitrogen, D3911, Carlsbad, CA, USA) were placed into neocortex of the whole brains at P7. Brains were stored in the dark at 37 °C for at least 4 wk to allow DiI diffusion. Brains were embedded in 2% low melt agarose and sectioned into 150 μm on vibratome. Sections were stained with Hoechst 33342 diluted 1/1 000 in PBS, then mounted with mounting medium and kept at 4 °C until the microscopic analysis.

### Western-blotting assay

Tissues or cells were lysed in RIPA buffer with 1 mM PMSF, 50 mM DTT, 1 mM EDTA, 1 mM NaF and protease inhibitor cocktail (Roche, 4693159001, Basel, Switzerland). After centrifuge, the protein extractions were separated by SDS-PAGE and followed by incubation with primary antibodies for 1 h at room temperature and 4 °C overnight. Then, secondary antibodies conjugated with horseradish peroxidase were added and incubated for 1 h. The bands were visualized by Chemiluminescence (Millipore, Billerica, MA, USA).

### RNA extraction and Quantitative real-time PCR

Total RNAs from E15.5 or E16.5 telencephalons were extracted by using Trizol Reagent (Invitrogen, 15596026, Carlsbad, CA, USA). RNA (1 μg) was reverse transcribed with GoStriptTM Reverse Transcription System (Promega, A5001, Madison, WI, USA). Quantitative real-time PCR reactions for target genes were performed using Hieff UNICON^®^ Universal Blue qPCR SYBR Green Master Mix. Gapdh was used as a normalization control for mRNA.

### In situ hybridization

In situ hybridization analyses were performed on 10 μm thick frozen sections according to standard procedures [79]. Digoxigenin-labelled antisense RNA probes were hybridized to brain sections at 65 °C for 14–16 h. Hybridized sections were washed, incubated with anti-digoxigenin-alkaline phosphatase (AP) antibody (1:1000) at 4 °C overnight, then washed and subjected to color reaction. Images were acquired by Leica Aperio VESA8 (Leica, Wetzlar, Germany). Please see the Key resources table for the details of all the materials used in this study.

### BrdU labelling assay

To analysis the fate and translocation of midline glia cells, pregnant females were given once injection of BrdU (i.p., 50 mg/kg body weight) at E14.5. The embryos were harvested at E16.5 and processed for immunohistochemistry.

### Quantification of cell number

To quantify the number of Sox9^+^ and/or BrdU^+^Sox9^+^ cells in the GW and/or IG region of *Tmco1*^+/+^ and *Tmco1*^−/−^ brains, a stereology-like counting method was performed by analyzing three coronal sections, 10 μm each, separated 20 μm one to each other at rostral, middle, and caudal position, respectively. Serial coronal frozen sections of the embryonic brains were cut by a cryostat, mounted on slides (three sections/slide) and stored at −80 °C. The “Middle” position of each brain was defined by the appearance of anterior commissure. Since the anterior commissure structures were not affected in the *Tmco1*^−/−^ brains, we use the morphology of anterior commissure to define the “Middle” position by bright field microscope. Then twelve sections (~120 μm, four slides) at E17.5, or nine sections (~90 μm, three slides) at E16.5 forward “Middle” was defined as the “Rostral”. The same distance backward “Middle” was defined as “Caudal”. Three sections at rostral/middle/caudal positions from each brain were selected, immunostained, and analyzed. A counting box of 200 × 150 μm was placed on immunostaining images of coronal sections and three rostro-caudal positions were used to quantify numbers of SOX9^+^ and/or BrdU^+^Sox9^+^ cells in the GW and IG compartments as reported previously [15, 45]. For each genotype, three or four embryonic brains were analyzed.

### Cell culture

HeLa cells were cultured in Dulbecco’s modified Eagle’s medium (DMEM) with 10% fetal bovine serum (FBS) at 37 °C in a 5% CO_2_ atmosphere. All the cell cultures were tested negative for mycoplasma contamination.

Primary cortical neurons were isolated from C57BL/6 J mice at embryonic day 18 as described previously [80]. The cells were maintained in neurobasal medium supplemented with 2% B27, 2 mM Glutamax, 1% penicillin-streptomycin in a humidified atmosphere of 5% CO_2_ at 37 °C. For the immunostaining experiments, isolated cortical neurons were plated into the petri confocal dishes pre-coated with Poly-L-Ornithine along with laminin, and then the medium was exchanged with fresh medium 6 h after plating to remove the tissue fragments. Half of the culture medium volume in each dish was changed with fresh medium every 2 days.

### Neurite outgrowth assay from cortical neurons

At DIV3 or DIV7, neurons were fixed with 4% PFA in PBS for 10 min at room temperature (RT) and then washed three times with PBS, followed by permeabilization with 0.25% TritonX-100 in PBS for 15 min at RT. The cells were then washed three times with PBS. After blocking with 5% bovine serum albumin in PBS for 1 h at 37 °C, the cells were incubated with the rabbit antibody against TuJ1 overnight at 4 °C. The next day, after 30 min rewarming, cells were washed three times with PBS and incubated for 2 h at RT with an Alexa 488-conjucated anti rabbit secondary antibody. Nuclei were counterstained with Hoechst 33342. Next, cells were washed three times with PBS. Images were acquired through Andor Dragonfly 505.

Two parameters of the neurite outgrowth of TuJ1^+^ cortical neurons were analyzed by Image J: the length of the longest neurites per neuron and the number of branch points per neuron. Neurite length was assessed by measuring the length from one cell body to the end of neuritis. To determine the longest neurite per neuron, multiple neurites per neuron were measured and the longest of those measure was selected. The number of neurite branches from cortical neurons was assessed using a Sholl analysis plugin to Image J. The concentric circles with a radius of 10 μm were drawn around the cell body, and the number of neurite intersections with each circle was automatically counted by ImageJ.

### Intracellular Ca^2+^ measurement

Ca^2+^ measurement was performed as described previously [31]. The cells attached on coated confocal dishes were loaded with 4 μM of calcium indicator Fura2-AM (Invitrogen, F1221, Carlsbad, CA, USA) and 0.02% pluronic F-127 (Invitrogen, P3000MP, Carlsbad, CA, USA) for 40 min at 37 °C in Hank’s Balanced Salt Solution (HBSS): 140 mM NaCl, 5 mM KCl, 10 mM Glucose, 10 mM HEPES, 2 mM CaCl_2_, 1 mM MgCl_2_, pH 7.4. After several washes with HBSS, cells were stayed for extra 10 min in HBSS buffer to allow de-esterification. Ca^2+^ measurements were done by Nikon inverted microscope (Eclipse TiE) with a ×40 magnification oil-immersion objective by alternatively excitation with 340 nm and 380 nm every 3 s, and the Fura-2 images were captured and processed by Metamorph software (version 7.0). The images were background subtracted, and the Ca^2+^ level was determined by the 340/380 fluorescence ratio. FGF8b was adopted 50 ng/ml for HeLa WT and KD cells at 37 °C to trigger intracellular Ca^2+^ oscillations, so did FGF17.

### MEK inhibitor treatment

The MEK inhibitor––mirdametinib (PD0325901) was dissolved in DMSO at a concentration of 25 mg/ml and solubilized at 5 mg/ml in vehicle (4% DMSO, 30% PEG 300, 5% Tween 80). Mirdametinib was administered to pregnant females by intraperitoneal injection at a concentration of 3 mg/kg body weight daily from embryonic day 14.5 to 16.5 after fertilization. Another MEKi––selumetinib (AZD6244) was administered to the pregnant females intragastrically (i.g., 15 mg/kg) from embryonic day 13.5 to 16.5 daily. Embryos were collected at E17.5 and MEKi treated embryos were compared with controls. 2–3 months old pregnant females were used for all MEKi experiments.

### Statistical analysis

All experiments were successfully reproduced at least twice. Final number of biological replicates is clearly indicated in each figure legend. For all experiments, at least three biological or experimental replicates were analyzed. Sample size for each experiment is indicated in the corresponding figure legend. No data were excluded from the analyses. However, dead mice were excluded from assessment. No statistical calculation was performed to predetermine sample size. Mice were randomly assigned and used in experimental groups. Experimental groups were based on age and/or treatment. For animal experiments, cohort size was determined by experimental type, availability of animals, and using standard cohort sizes in our field ranging 7–12 mice/group for in vivo experiments. Blinding method was used for MEKi injection. The investigators were not blinded to group allocation during other experiments. Data are expressed as the mean and the statistical significance of differences between different groups is assessed using the two-tailed unpaired Student’s *t* test or ANOVA. Significance was set at *P* < 0.05, *P* < 0.01, *P* < 0.001. Specific *P* values are indicated in the figures. All statistical tests were performed by Graphpad Prism 8.

## Supplementary information

Supplementary files

Original western blots

Reproducibility Checklist

Supplemental figure 1

Supplemental figure 2

Supplemental figure 3

Supplemental figure 4

## Data Availability

Data are available upon reasonable request.
