# Supplementary material for: Ca2+ homeostasis maintained by TMCO1 underlies corpus callosum development via ERK signaling
Source: Cell Death Dis. 2022 Aug 4;13(8):674. doi: 10.1038/s41419-022-05131-x (PMC9352667; doi:10.1038/s41419-022-05131-x)
Supplement: Supplementary file 1 — Supplementary files [file 41419_2022_5131_MOESM1_ESM.docx]

**Ca^2+^ homeostasis maintained by TMCO1 underlies corpus callosum development via ERK signaling**

Ke-Yan Yang, Song Zhao, Haiping Feng, Jiaqi Shen, Yuwei Chen, Si-Tong Wang, Si-Jia Wang, Yu-Xin Zhang, Yun Wang, Caixia Guo, Hongmei Liu and Tie-Shan Tang

Corresponding author: : [guocx@big.ac.cn](mailto:guocx@big.ac.cn) (C.G.), [liuhongmei@ioz.ac.cn](mailto:liuhongmei@ioz.ac.cn) (H.L.), [tangtsh@ioz.ac.cn](mailto:tangtsh@ioz.ac.cn) (T.-S.T.)

**This PDF file includes:**

Supplementary text

Figure legends S1 to S4

Key resources table

**Supplementary Figure legends**

**Supplementary Figure 1. The expression pattern of TMCO1 during the period of corpus callosum formation. Related to Figure 1.**

(A) *In situ* hybridization for the expression of *Tmco1* in the telencephalon of *Tmco1^+/+^* embryos at E13.5, E15.5, E16.5 and E17.5. High magnification of the areas boxed are shown on the right. Scale bar, 200 μm. CP, cortical plate; IG, indusium griseum; GW, glia wedge; VZ/SVZ, ventricular zone/subventricular zone.

(B) Negative control for the *in situ* hybridization of *Tmco1* in the telencephalon of *Tmco1^+/+^* embryos. Scale bar, 1 000 μm.

(C) qRT-PCR analysis of *Tmco1* expression in the whole brains of *Tmco1*^+/+^ from E13.5 to P28. One-way ANOVA with Tukey’s correction for multiple comparisons.

(D) Western-blotting analysis of proteins extracted from whole telencephalon of *Tmco1*^+/+^ from E13.5 to P28. GAPDH is used as a loading control. Each band was quantified and normalized to GAPDH. The relative changing fold was underlined. n = 3. One-way ANOVA with Tukey’s correction for multiple comparisons.

(E) Western-blotting analysis of proteins extracted from E16.5 whole telencephalon of *Tmco1*^+/+^ and *Tmco1*^-/-^. GAPDH is used as a loading control. *Tmco1*^+/+^ group, n = 3. Two-tailed unpaired Student’s *t* test.

**Supplementary Figure 2. Normal specification of callosal projection neurons,** **lamination in *Tmco1*^+/+^ and *Tmco1*^-/-^ mice and neurite outgrowth in cortical neurons *in* *vitro*. Related to Figure 2.**

(A) Immunofluorescence of neocortex for three layer-specific makers (CTIP2, SATB2 and TRB1) in E17.5 brains of *Tmco1*^+/+^ and *Tmco1*^-/-^. Scale bar, 100 μm. H, Hoechst.

(B) Quantitative analysis of neocortical layer thickness revealed no significance between *Tmco1*^-/-^ and *Tmco1*^+/+^. The UP layers were defined by the SATB2-positve cells upon the CTIP2-positive layers. The DP layers were defined by the CTIP2-positive cells. *Tmco1*^+/+^ group, n = 8; *Tmco1*^-/-^ group, n = 10. Two-tailed unpaired Student’s *t* test.

(C) The H&E staining of coronal sections of *Tmco1*^+/+^ and *Tmco1*^-/-^ brains at E14.5, E15.5 and E16.5 during corpus callosum formation. Arrowhead, the axons reached at the midline at E15.5 in both *Tmco1*^+/+^ and *Tmco1*^-/-^ mice. Arrow, the affected corpus callosum at E16.5 of *Tmco1*^-/-^ mice. Scale bar, 500 μm.

(D) The neurite outgrowth of E18 cortical neurons at DIV3. Left panel, images of cultured cortical neurons stained with anti-TuJ1 antibody. Scale bar, 100 μm. Right panel, quantification of the length of the longest neurite of cortical neurons. n = 80 cells at DIV3. Two-tailed unpaired Student’s *t* test.

(E) The neurite outgrowth of E18 cortical neurons at DIV7. Left panel, images of cultured cortical neurons stained with anti-TuJ1 antibody. Scale bar, 100 μm. Right panel, quantification of the length of the longest neurite of cortical neurons. n = 30 cells at DIV7. Two-tailed unpaired Student’s *t* test.

(F) Quantification of the neurite complexity of Tuj1-positive neurons by Sholl analysis of neurite intersections at DIV7.

**Supplementary Figure 3. Characterization of the expression patterns of axon guidance factors and receptors during corpus callosum development. Related to Figure2.**

(A) *In situ* hybridization for the expression of *Netrin1, an* axon guidance factor, together with its co-response receptor - *DCC* and *Unc5c* in the telencephalon of *Tmco1^+/+^ and Tmco1*^-/-^ embryos at E15.5 and E16.5. Scale bar, 500 μm.

(B) Left, *in situ* hybridization for the expression of axon guidance factor *Sema3C* in the telencephalon of *Tmco1^+/+^ and Tmco1*^-/-^ embryos at E15.5. Right, representative images for co-staining of Sema3A and its receptor – Nrp1(Neuropilin 1) in the telencephalon of *Tmco1^+/+^ and Tmco1*^-/-^ embryos at E15.5. Scale bar, 500 μm.

(C) *In situ* hybridization for the expression of axon guidance factor *EfnB3* and receptors – *EphA1*, *EphB1*, *EphB2* and *EphB3* in the telencephalon of *Tmco1^+/+^ and Tmco1^-/-^* embryos at E16.5. Scale bar, 500 μm.

**Supplementary Figure 4. Both mirdametinib and selumetinib are effective to ameliorate the AgCC in *Tmco1* mutants. Related to Figure 5.**

(A) Nissl staining of the coronal sections in the *Tmco1*^+/+^ and *Tmco1*^-/-^ brains after the mirdametinib treatments. Scale bar, 500 μm.

(B) The timeline of selumetinib treatment (i.g., 15 mg/kg), which was administrated to pregnant females daily from E13.5 to E16.5 and harvested at E17.5.

(C) Nissl staining of the coronal sections in the *Tmco1*^+/+^ and *Tmco1*^-/-^ brains treated with/without selumetinib. Scale bar, 500 μm.

(D) Quantification of the number of *Tmco1*^-/-^ embryos showing normal or AgCC phenotypes with/without selumetinib treatment. Untreated pregnant females, n = 8. Selumetinib-treated pregnant females, n = 7.

(E) Quantification of the thickness of corpus callosum after MEKi treatments. *Tmco1*^+/+^ vehicle group, n = 3; *Tmco1*^-/-^ vehicle group, n = 4; *Tmco1*^+/+^ mirdametinib group, n = 4; *Tmco1*^-/-^ mirdametinib group, n = 9; *Tmco1*^+/+^ selumetinib group, n = 6; *Tmco1*^-/-^ selumetinib group, n = 9. One-way ANOVA with Tukey’s correction for multiple comparisons.

**Key resources table**

| REAGENT or RESOURCE | SOURCE | IDENTIFIER |
| --- | --- | --- |
| Antibodies | | |
| Rat Anti-CTIP2 Monoclonal Antibody | Abcam, Cambridge, MA, USA | Cat#ab18465; RRID: AB_2064130 |
| Rat Anti-BrdU Monoclonal Antibody | Abcam, Cambridge, MA, USA | Cat#ab6326; RRID: AB_305426 |
| Rabbit Anti-SATB2 Polyclonal Antibody | Abcam, Cambridge, MA, USA | Cat#ab34735; RRID:AB_2301417 |
| Rabbit Anti-TBR1 Polyclonal Antibody | Abcam, Cambridge, MA, USA | Cat#ab31940; RRID: AB_2200219 |
| Rabbit Anti-Tuj1 Polyclonal Antibody | Abcam, Cambridge, MA, USA | Cat#ab18207; RRID: AB_444319 |
| Rat Anti-Neural Cell Adhesion Molecule L1 (L1CAM) Monoclonal Antibody | Millipore, Billerica, MA, USA | Cat#MAB5272; RRID: AB_2133200 |
| Rabbit Anti-ZIC-2 Polyclonal Antibody | Millipore, Billerica, MA, USA | Cat#AB15392; RRID: AB_1977437 |
| Rabbit Anti-SOX-9 Polyclonal Antibody | Millipore, Billerica, MA, USA | Cat#AB5535; RRID: AB_2239761 |
| Goat Anti-GLI-3 Polyclonal Antibody | R&D Systems, Minneapolis, MN, USA | Cat#AF3690; RRID: AB_2232499 |
| Human/Mouse Anti-FGF-8 Monoclonal Antibody | R&D Systems, Minneapolis, MN, USA | Cat#MAB323; RRID: AB_2102956 |
| Rabbit Anti-NF-1A Polyclonal Antibody | Active Motif, Carlsbad, CA, USA | Cat#39397; RRID: AB_2314931 |
| Rabbit-Phospho-p44/42 MAPK (Erk1/2) (Thr202/Tyr204) Monoclonal Antibody | Cell Signaling Technology, Danvers, CA, USA | Cat#4370S; RRID: AB_2315112 |
| Rabbit-p44/42 MAPK (Erk1/2) (137F5) Monoclonal Antibody | Cell Signaling Technology, Danvers, CA, USA | Cat#4695S; RRID: AB_390779 |
| Sheep-anti-Digoxigenin- AP Fab fragments antibody | Roche, Basel, Switzerland | Cat#11093274910; RRID: AB_514497 |
| Rabbit-FGF17 Polyclonal Antibody | Proteintech, Wuhan, Hubei | Cat#25314-1-AP; RRID: AB_2880025 |
| Chemicals, peptides, and recombinant proteins | | |
| Recombinant Human FGF-17 Protein | R&D Systems, Minneapolis, MN,USA | Cat#319-FG-025/CF |
| Recombinant Human/Mouse FGF-8b Protein | R&D Systems, Minneapolis, MN,USA | Cat#423-F8-025/CF |
| Mirdametinib (PD0325901) | TOPSCIENCE, Boston, MA, USA | Cat#T6189 |
| Selumetinib (AZD6244) | Selleck Chemicals, Houston, TX,USA | Cat#S1008 |
| Experimental models: Cell lines | | |
| HeLa cells | ATCC, Manassas, VA, USA |  |
|  |  |  |
| Experimental models: Organisms/strains | | |
| *Tmco1*^+/-^ mice; Background: C57BL/6J | Shanghai Model Organisms Center, Inc. |  |
|  |  |  |
| Oligonucleotides | | |
| mouse *Tmco1* (qRT-PCR) | Integrated DNA Technologies | Forward: 5’-TCATCGTCTTTATCTCCGTGTGC-3’  Reverse: 5’-GGATTTCATCCGTACCATTGACA-3’ |
| mouse *Fgf8* (qRT-PCR) | Integrated DNA Technologies | Forward: 5’-GAGACCGATACTTTTGGAAGCA-3’  Reverse: 5’-TCTCTGTGAATACGCAGTCCT-3’ |
| mouse *Fgf17* (qRT-PCR) | Integrated DNA Technologies | Forward: 5’-GTGCTTGCAGCTATTGATTCTCT-3’  Reverse: 5’-GGTCCCTCACGTACTGGTTAAAA-3’ |
| mouse *Tmco1-*probe-exp (*In situ* hybridization) | Integrated DNA Technologies | Forward: 5’-GATGAAGGAGCAGTCCGTGG-3’  Reverse: 5’-ACGCTGCTCATCGTCTTTAT-3’ |
| mouse *Tmco1*-probe-ctrl (*In situ* hybridization) | Integrated DNA Technologies | Forward: 5’-ACGCTGCTCATCGTCTTTAT-3’  Reverse: 5’-GATGAAGGAGCAGTCCGTGG-3’ |
| mouse *Fgf8*-probe (*In situ* hybridization) | Integrated DNA Technologies | Forward: 5’-GCTTCCCCTTCTTGTTCATGC-3’  Reverse: 5’-ACTTGCTGGTTCTCTGCCTC-3’ |
| mouse *Fgf17*-probe (*In situ* hybridization) | Integrated DNA Technologies | Forward: 5’-TTCTGCCTTTCAGCGTGGTT-3’  Reverse: 5’-GCGGCAAATCCGTGAATACC-3’ |
| mouse *Slit2*-probe (*In situ* hybridization) | Integrated DNA Technologies | Forward: 5’-TTCAGAGAGCGCAGTCCTTG-3’  Reverse: 5’-TCGACTCCACTCGAACAACT-3’ |
| Recombinant DNA | | |
| pBluescript II SK(+) | GenStar, BeiJing | N/A |
|  |  |  |
| Software and algorithms | | |
| GraphPad Prism | GraphPad, La Jolla, CA, USA | Version: 8 |
| LAS X | Leica, Wetzlar, Germany | Version: 3.5.7 |
| ImageJ | ImageJ, NIH, Bethesda, MD, USA | Version: 1.53c |
| Other | | |
|  |  |  |
