# Supplementary material for: Ca2+ homeostasis maintained by TMCO1 underlies corpus callosum development via ERK signaling
Source: Cell Death Dis. 2022 Aug 4;13(8):674. doi: 10.1038/s41419-022-05131-x (PMC9352667; doi:10.1038/s41419-022-05131-x)

Original data of WB

Figure 3E. Western-blotting analysis of FGF8 extracted from E16.5 rostral-medial brain of *Tmco1*^+/+^ and *Tmco1*^-/-^.

TMCO1


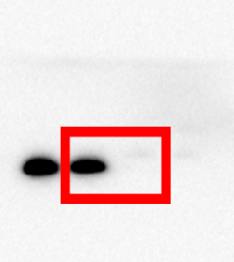


FGF8


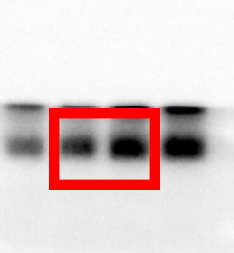


GAPDH


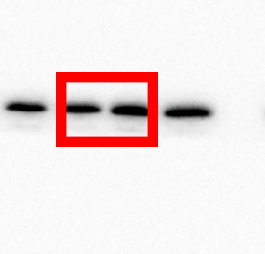


Figure 3F Western-blotting analysis of FGF17 extracted from E16.5 rostral-medial brain of *Tmco1*^+/+^ and *Tmco1*^-/-^.

FGF17


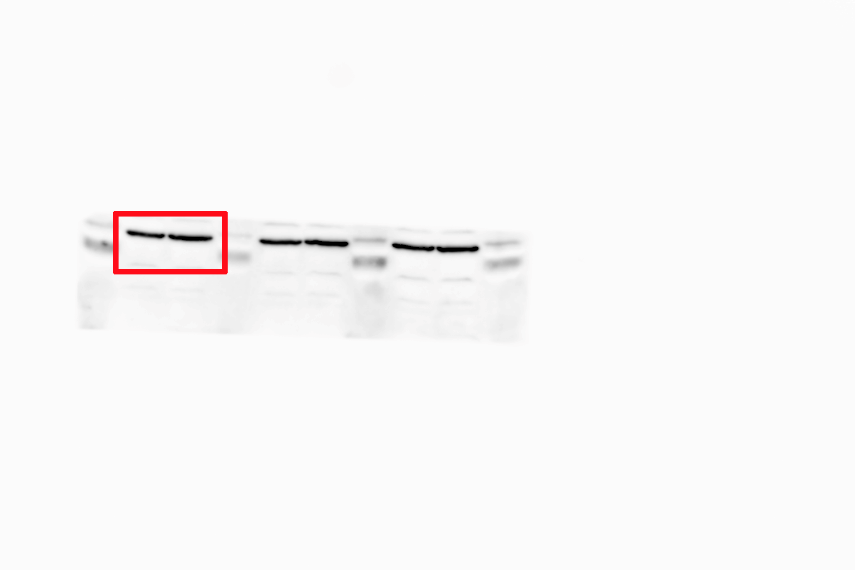


Actin


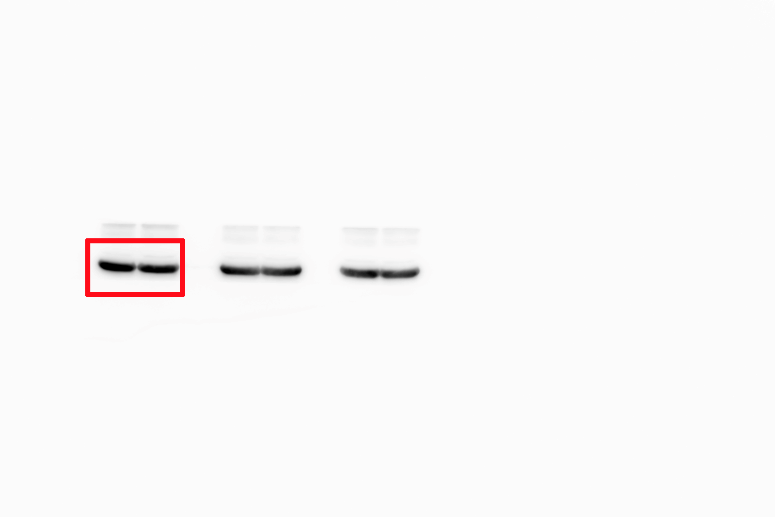


Figure 3I. Western-blotting analysis of proteins extracted from E16.5 whole telencephalons of *Tmco1*^+/+^ and *Tmco1*^-/-^.

P-ERK


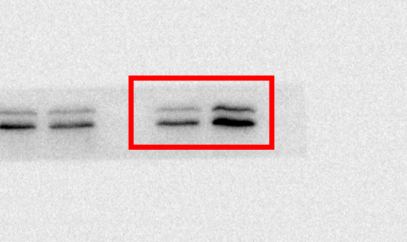


ERK


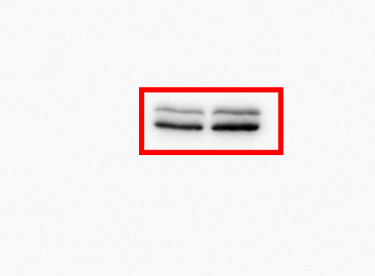


TMCO1


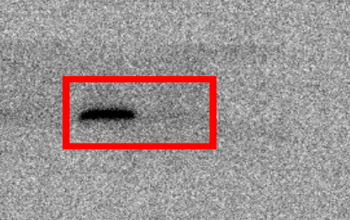


GAPDH


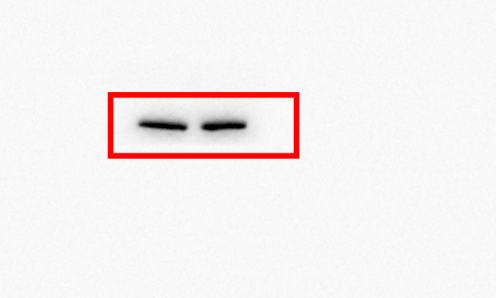


Figure 4D. Western-blotting analysis of proteins extracted from WT or *Tmco1*-KD HeLa cells treated with/without 50 μM BAPTA-AM.

P-ERK


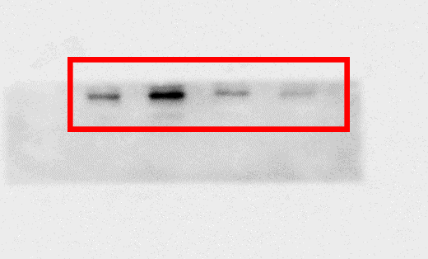


ERK


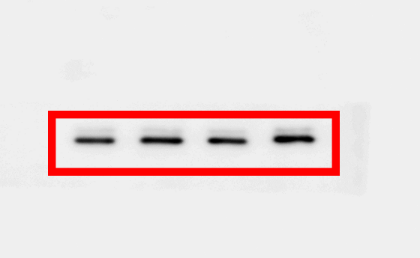


TMCO1


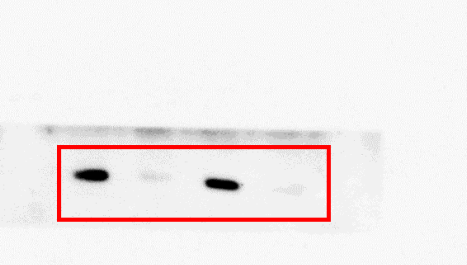


Tubulin


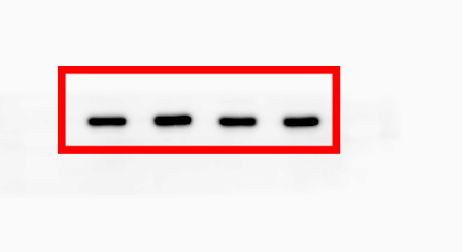


Figure 4E. Western-blotting analysis of proteins extracted from WT or *Tmco1*-KD HeLa cells treated with FGF8b together with/without 50 μM BAPTA-AM.

P-ERK


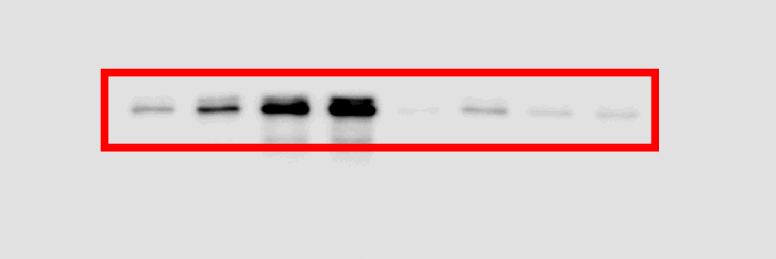


ERK


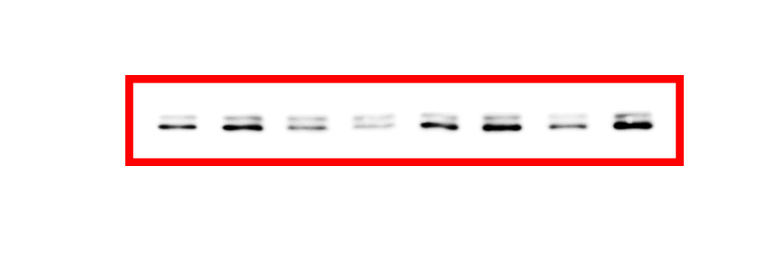


TMCO1


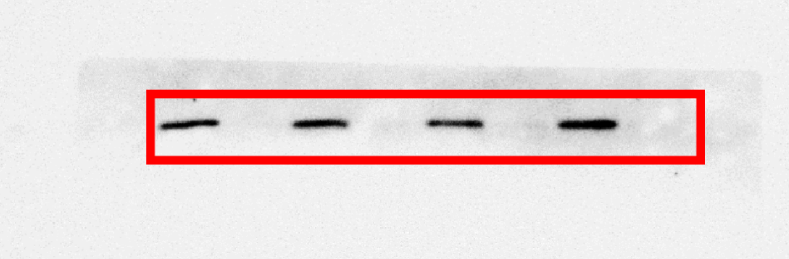


Tubulin


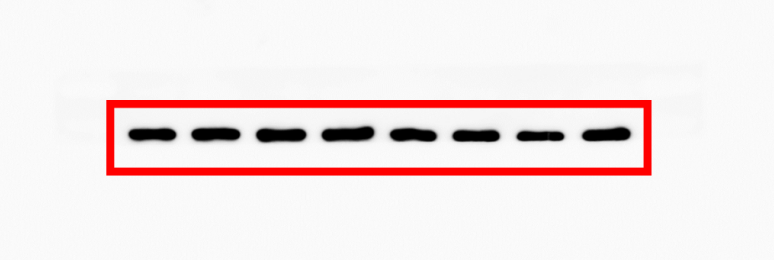


Figure 4F. Western-blotting analysis of proteins extracted from WT or *Tmco1*-KD HeLa cells treated with FGF17 together with/without 50 μM BAPTA-AM.

P-ERK


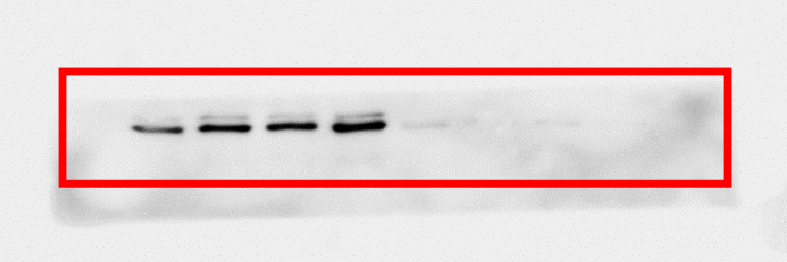


ERK


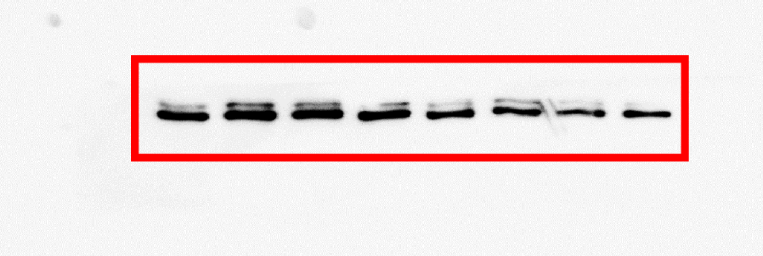


TMCO1


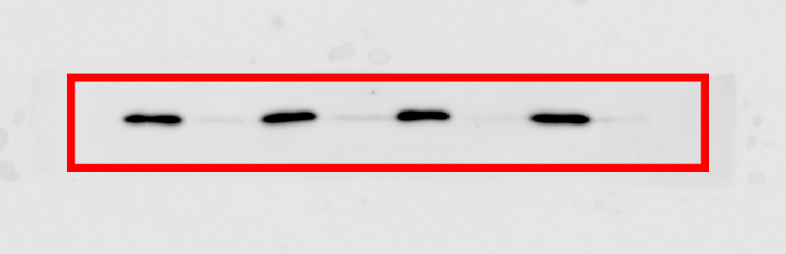


Tubulin


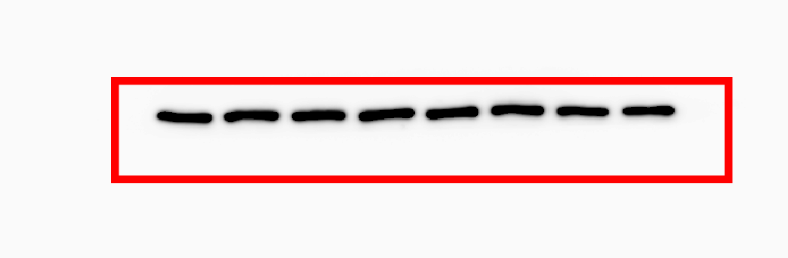


Supplementary Figure 1D. Western-blotting analysis of proteins extracted from whole telencephalon of *Tmco1*^+/+^ from E13.5 to P28.

TMCO1


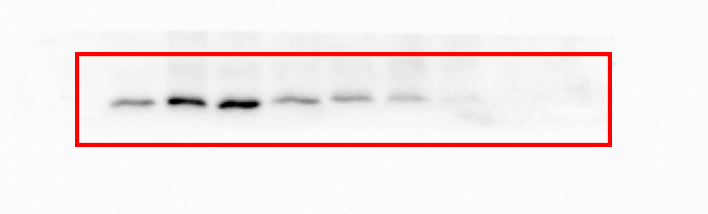


GAPDH


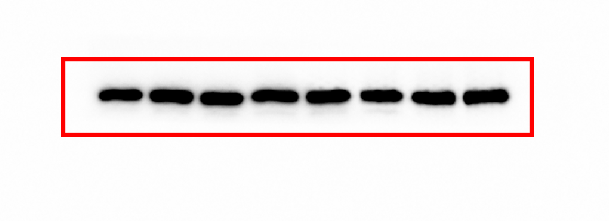


Supplementary Figure 1E Western-blotting analysis of proteins extracted from E16.5 whole telencephalon of *Tmco1*^+/+^ and *Tmco1*^-/-^

TMCO1


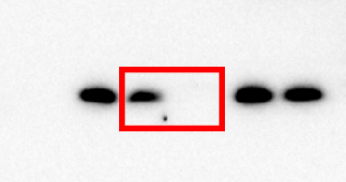


GAPDH


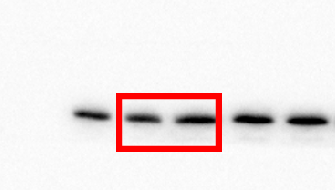

Supplement: Supplementary file 2 — Original western blots [file 41419_2022_5131_MOESM2_ESM.docx]
