# Supplementary figures and images for: Ca2+ homeostasis maintained by TMCO1 underlies corpus callosum development via ERK signaling
Source: Cell Death Dis. 2022 Aug 4;13(8):674. doi: 10.1038/s41419-022-05131-x (PMC9352667; doi:10.1038/s41419-022-05131-x)

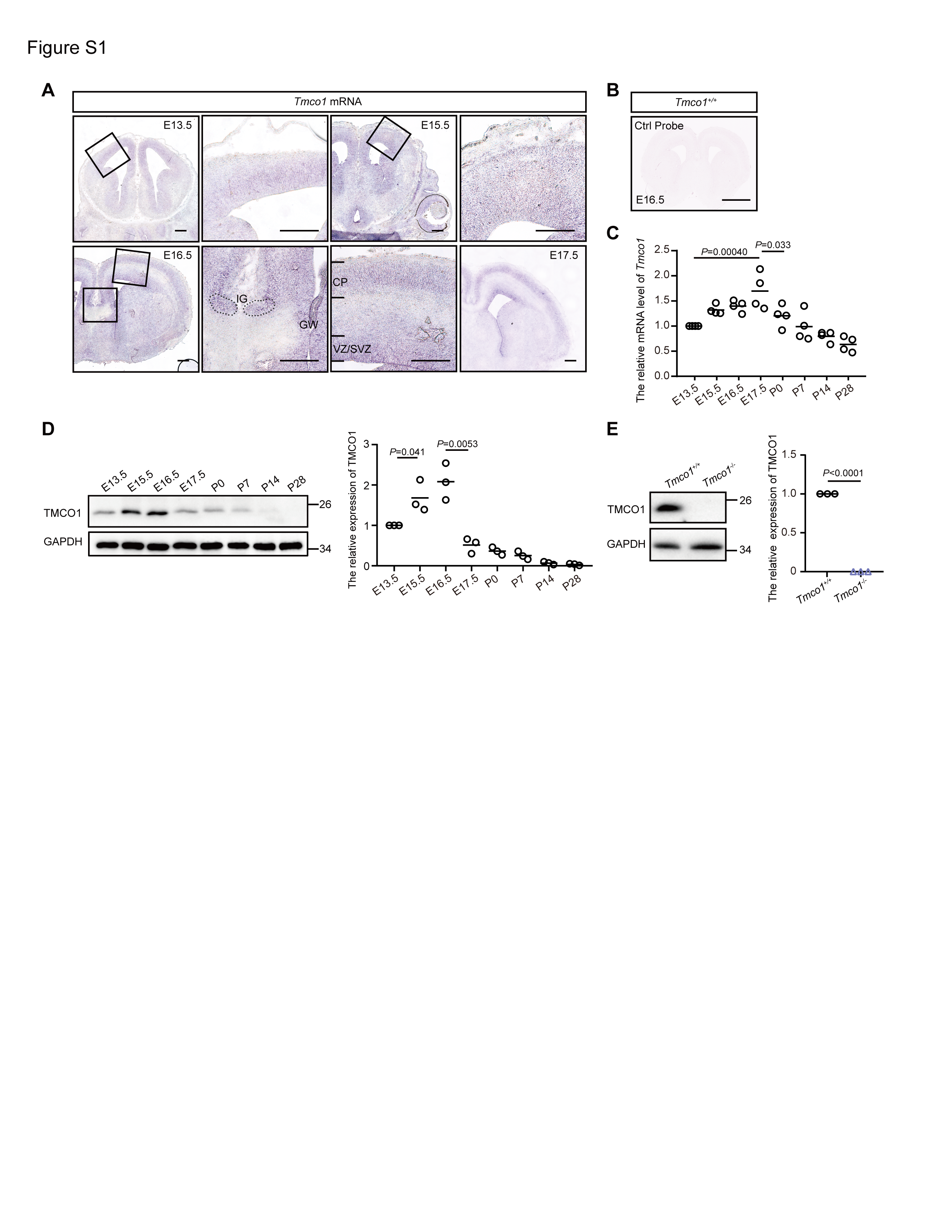

Supplement: Supplementary file 4 — Supplemental figure 1 [file 41419_2022_5131_MOESM4_ESM.png]

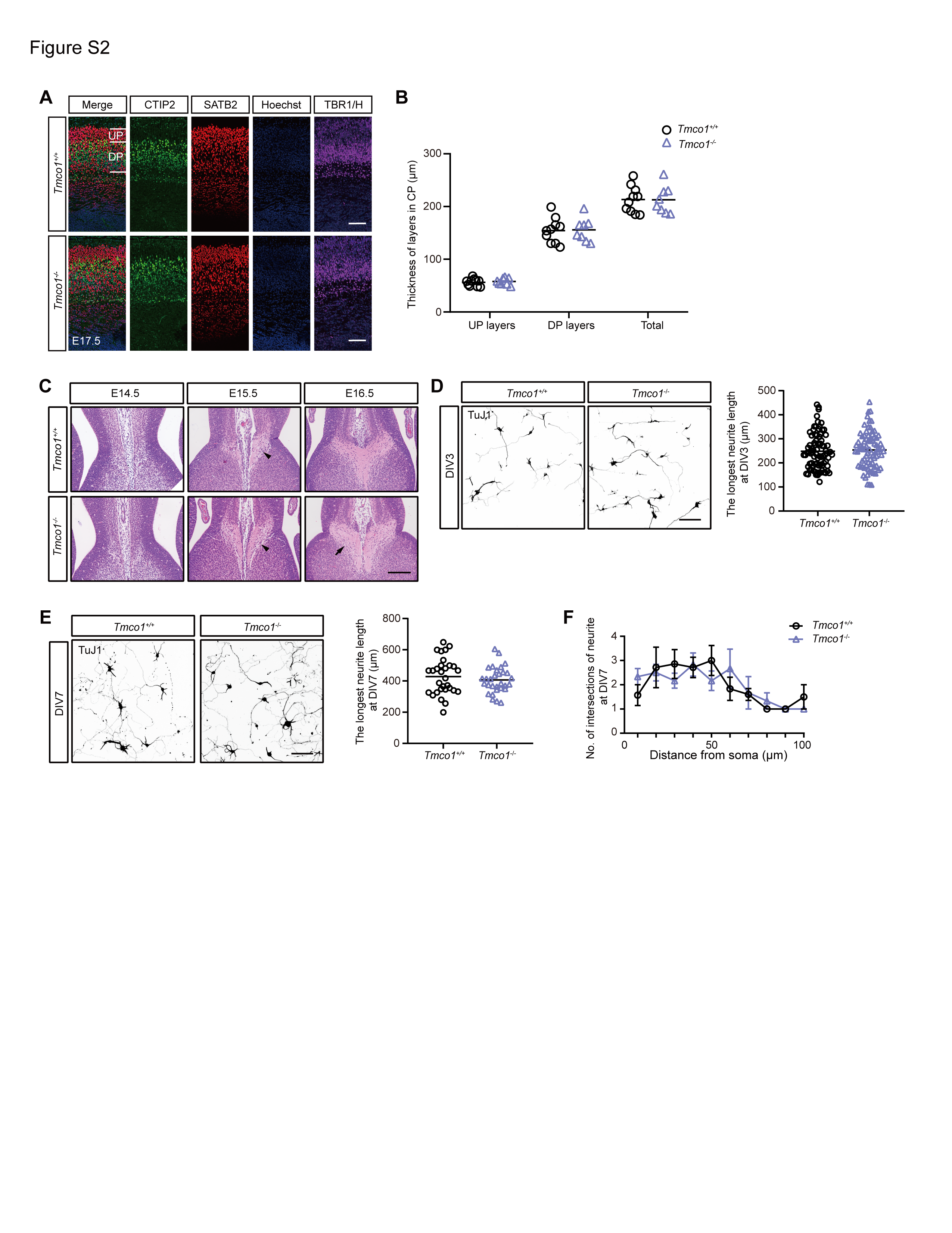

Supplement: Supplementary file 5 — Supplemental figure 2 [file 41419_2022_5131_MOESM5_ESM.png]

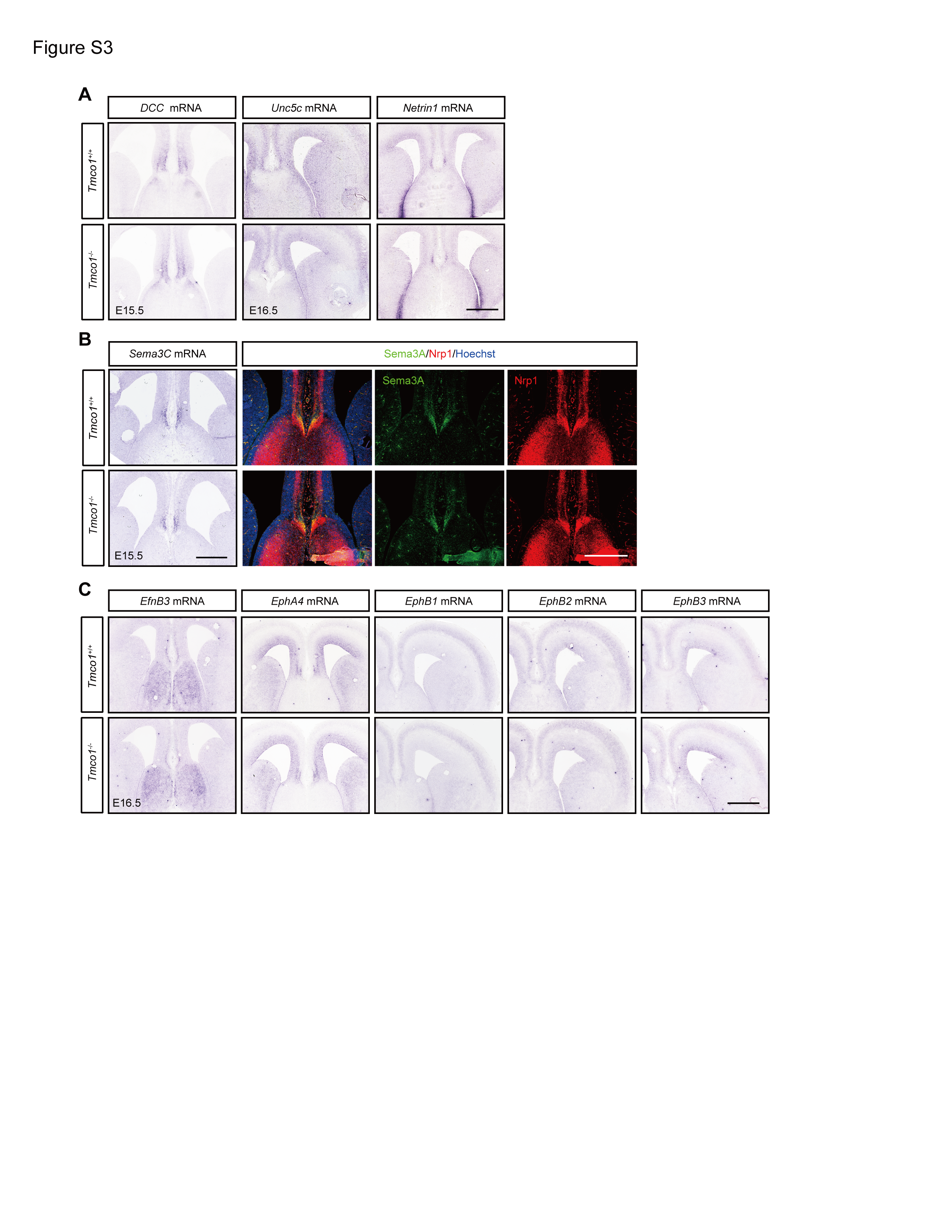

Supplement: Supplementary file 6 — Supplemental figure 3 [file 41419_2022_5131_MOESM6_ESM.png]

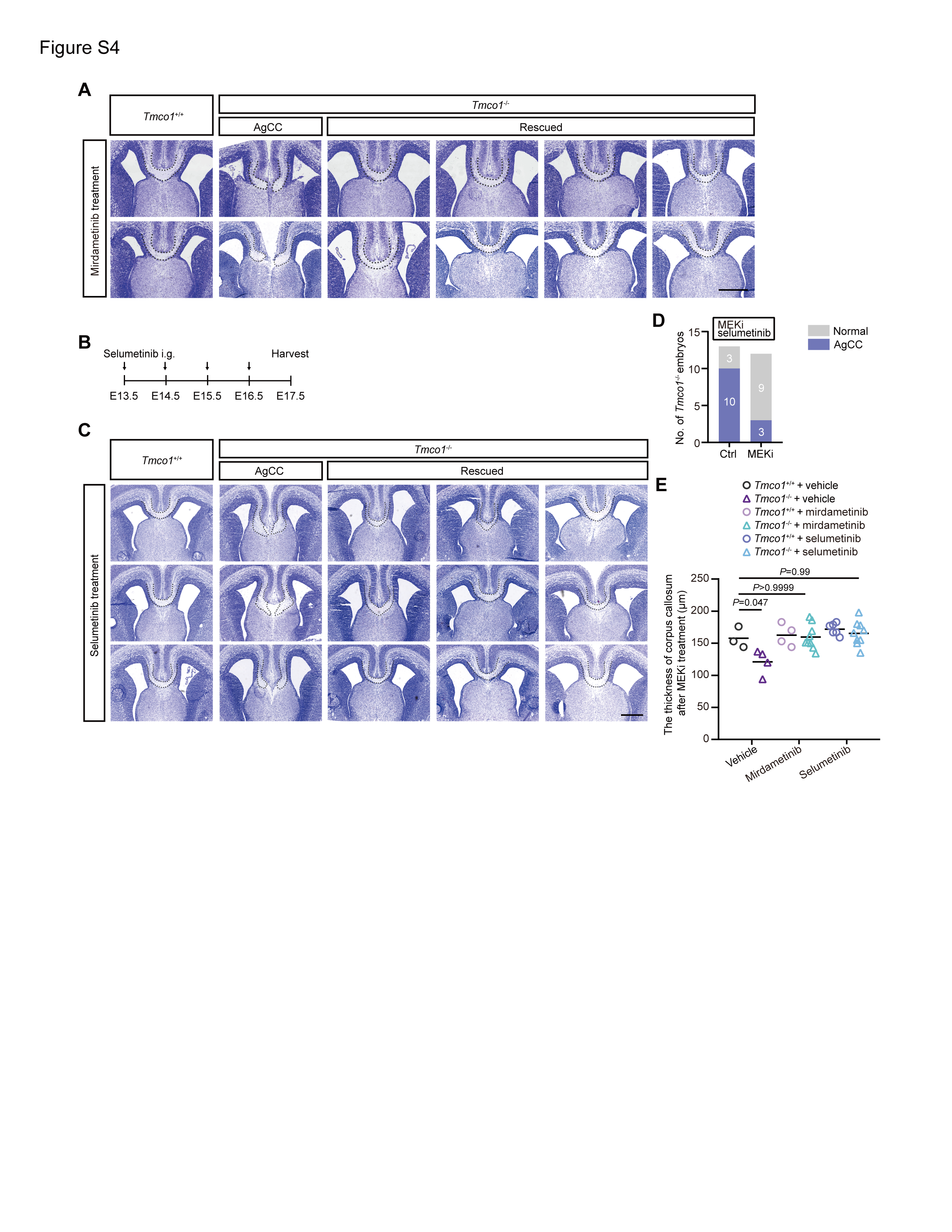

Supplement: Supplementary file 7 — Supplemental figure 4 [file 41419_2022_5131_MOESM7_ESM.png]
